# Severe Eczema in Infancy Can Predict Asthma Development. A Prospective Study to the Age of 10 Years

**DOI:** 10.1371/journal.pone.0099609

**Published:** 2014-06-10

**Authors:** Marie Ekbäck, Michaela Tedner, Irene Devenney, Göran Oldaeus, Gunilla Norrman, Leif Strömberg, Karin Fälth-Magnusson

**Affiliations:** 1 Division of Pediatrics, Department of Clinical and Experimental Medicine, Faculty of Health Sciences, Linköping University and Department of Pediatrics, County Council of Östergötland, Linköping, Sweden; 2 Pediatric Clinic, Täby, Stockholm, Sweden; 3 Pediatric Clinic, County Hospital Ryhov, Jönköping, Sweden; 4 Pediatric Clinic, Hudiksvall, Sweden; 5 Department of Pediatrics in Norrköping, County Council of Östergötland, Norrköping, Sweden; Centre de Recherche Public de la Santé (CRP-Santé), Luxembourg

## Abstract

**Background:**

Children with atopic eczema in infancy often develop allergic rhinoconjunctivitis and asthma, but the term “atopic march” has been questioned as the relations between atopic disorders seem more complicated than one condition progressing into another.

**Objective:**

In this prospective multicenter study we followed children with eczema from infancy to the age of 10 years focusing on sensitization to allergens, severity of eczema and development of allergic airway symptoms at 4.5 and 10 years of age.

**Methods:**

On inclusion, 123 children were examined. Hanifin-Rajka criteria and SCORAD index were used to describe the eczema. Episodes of wheezing were registered, skin prick tests and IgE tests were conducted and questionnaires were filled out. Procedures were repeated at 4.5 and 10 years of age with additional examinations for ARC and asthma.

**Results:**

94 out of 123 completed the entire study. High SCORAD points on inclusion were correlated with the risk of developing ARC, (B = 9.86, P = 0.01) and asthma, (B = 10.17, P = 0.01). For infants with eczema and wheezing at the first visit, the OR for developing asthma was 4.05(P = 0.01). ARC at 4.5 years of age resulted in an OR of 11.28(P = 0.00) for asthma development at 10 years.

**Conclusion:**

This study indicates that infant eczema with high SCORAD points is associated with an increased risk of asthma at 10 years of age. Children with eczema and wheezing episodes during infancy are more likely to develop asthma than are infants with eczema alone. Eczema in infancy combined with early onset of ARC seems to indicate a more severe allergic disease, which often leads to asthma development. The progression from eczema in infancy to ARC at an early age and asthma later in childhood shown in this study supports the relevance of the term “atopic march”, at least in more severe allergic disease.

## Introduction

### Background

Children with an atopic constitution are at risk of developing allergic symptoms, such as atopic eczema, food allergy, allergic rhinoconjunctivitis (ARC) and asthma [1]–[3], with multiple factors influencing these immune responses. Especially in countries with a western life style the prevalence of allergy in childhood has increased remarkably in recent decades [2], [4], [5]. The “atopic march” is a term that has been used to describe the progression from atopic eczema and food allergy during infancy to ARC and subsequently to asthma later in childhood [6]–[9]. Previous studies have shown that children with atopic eczema or those sensitized to allergens in early childhood more often develop ARC and asthma [8], [10]–[13]. However, the concept of “atopic march” has been questioned. The relations between the allergic disorders seem to be much more complicated than that of one condition progressing into another [7], [8].

Wheezing during infancy is common, but is not necessarily caused by asthma. Epidemiological studies have described different phenotypes of asthma [3], [8], [14], [15], many of which are transient conditions with low risk of asthma and allergy later in life. However, 30–50% of the children involved have been reported to develop persistent asthma, especially if they are sensitized [2], [3], [16].

Several environmental factors have been associated with asthma in epidemiological studies, for example sensitization to aeroallergens early in life, exposure to pets, maternal diet during pregnancy and lactation, breastfeeding, exposure to tobacco smoke during pregnancy and early in life, viral infections, a disturbed balance of microbes, and psychosocial factors [3], [17]. Differences in opinion make it controversial to give advice concerning pets and maternal diet. Breastfeeding has been reported to decrease wheezing associated with respiratory infections in early childhood, but there is not enough evidence as to whether it prevents asthma [3], [18].

A better understanding of the process of developing allergic airway symptoms, including early warning signs, would be of great value. This could help to identify children at high risk of developing asthma and thereby enable early intervention and treatment and perhaps even aid prevention.

### Aim

The aim of the present study was to follow infants with eczema and suspected food allergy over time, focusing on sensitization to allergens, severity of eczema and the development of allergic airway symptoms at 4.5 and 10 years of age. Furthermore, we wanted to identify any early signs that could be associated with a higher risk of developing allergic airway symptoms.

## Methods

### Ethics statement

The study was approved by the Human Research Ethics Committees at the Faculty of Health Sciences Linköping University and at the Medical Faculty at Uppsala University. Written informed consent was obtained from the children's parents at all ages and from the children at 10 years of age.

### Methods

The study population was 123 children (71 boys and 52 girls) with eczema and suspected food allergy recruited to the study from June 1999 to September 2001. They were all under two years of age (median 6 months, range 1–23) and had been referred from their primary care physician to the pediatric clinics in Linköping, Jönköping, Norrköping and Hudiksvall [19].

Factors studied on inclusion and at follow-up examinations are presented in Table 1. At the first visit, the patients were examined by a pediatrician. Questionnaires regarding other atopic manifestations, family history, environmental factors and nutrition were completed by the parents. The diagnostic criteria proposed by Hanifin and Rajka were used to diagnose atopic dermatitis [20]. The severity of eczema was evaluated by a standardized method, “The Severity Scoring of Atopic Dermatitis” (SCORAD). SCORAD was used to classify eczema as mild (≤25 points), moderate (26–50 points) or severe (>50 points) [21]. Standardized skin prick tests (SPT) were performed in a standardized way [22]; a mean wheal diameter of three mm was used as the lower limit for positivity as recommended in the EAACI position paper [22], [23]. The SPTs and the SCORAD were assessed by experienced allergy nurses. All children were recommended treatment with emollients and/or topical steroids. Children with a positive SPT were recommended a temporary elimination diet. For food allergic children, milk and eggs were reintroduced in either open standardized or double-blind placebo-controlled oral food challenges when SPT was ≤10 mm and SCORAD ≤25points [19]. Six weeks after inclusion, the patients were reexamined by the same nurse using SCORAD and SPT. Venous blood samples were obtained on inclusion, at the six-week follow-up, at 4.5 and 10 years of age. IgE was analyzed with UniCAP, as previously described [24].

**Table 1:** An overview of examinations and tests performed on the children with eczema on inclusion, at 4.5 years and at 10 years.

	Inclusion	4,5 years	10 years
Physical examination	X	X	X
SCORAD	X	X	X
Hanifin-Rajka	X	X	X
SPT	X	X	X
IgE	X	X	X
Questionnaries	X	X	X
Wheezing episodes	X		
ARC		X	X
Asthma		X	X
Spirometry			X
Exhaled NO			X

The patients were reexamined by a pediatrician at 4.5 years and 10 years of age. Persisting eczema was assessed using Hanifin-Rajka criteria, severity was graded according to SCORAD at both visits, and venous blood samples were obtained. SPTs were performed for foods and aeroallergens (Table 1). Questionnaires enquiring about other allergic manifestations and tolerance to food were completed. At 10 years of age, lung function tests were performed. A standardized spirometric test combined with a standardized exercise test was performed. It included a period of 6 minutes intense running to increase the pulse rate to 160 beats per minute. Heart rate and oxygen saturation were documented. Spirometric values were measured before and immediately after running, after 10 minutes rest, and 15 minutes after inhalation of 1 mg terbutalin, inhaled from a multi-dose powder inhaler. Exhaled nitric oxide was measured with NIOX MINO (Aerocrine, Solna, Sweden) before and directly after the exercise period.

After the final examination at 10 years of age, the patients were divided into three groups. The first group was patients with no persisting allergic airway problems with or without eczema, the second group was patients suffering from ARC, and the third group was patients suffering from asthma. Patients with both ARC and asthma at 10 years were included in the asthma group.

The diagnostic criterion used for asthma in infants and preschool children in this study was any episode of obstructive airway symptoms in combination with IgE-mediated eczema, as recommended by the Swedish National Pediatric Allergy Group [25].

At the final visit for the 10 year-olds, an increase in FEV1 ≥10% after inhaling salbutamol or a reduction in FEV1 ≥10% of baseline levels after effort was considered diagnostic for asthma, as were patients previously diagnosed as fulfilling the criteria for asthma [26], [27]. ARC was defined as symptoms of rhinitis or conjunctivitis after exposure to allergens, correlating to a positive SPT or a positive test for specific IgE. Sensitization was defined as either a positive SPT or specific IgE values ≥0.35kUA/l [24].

### Statistics

**Figure 1:**
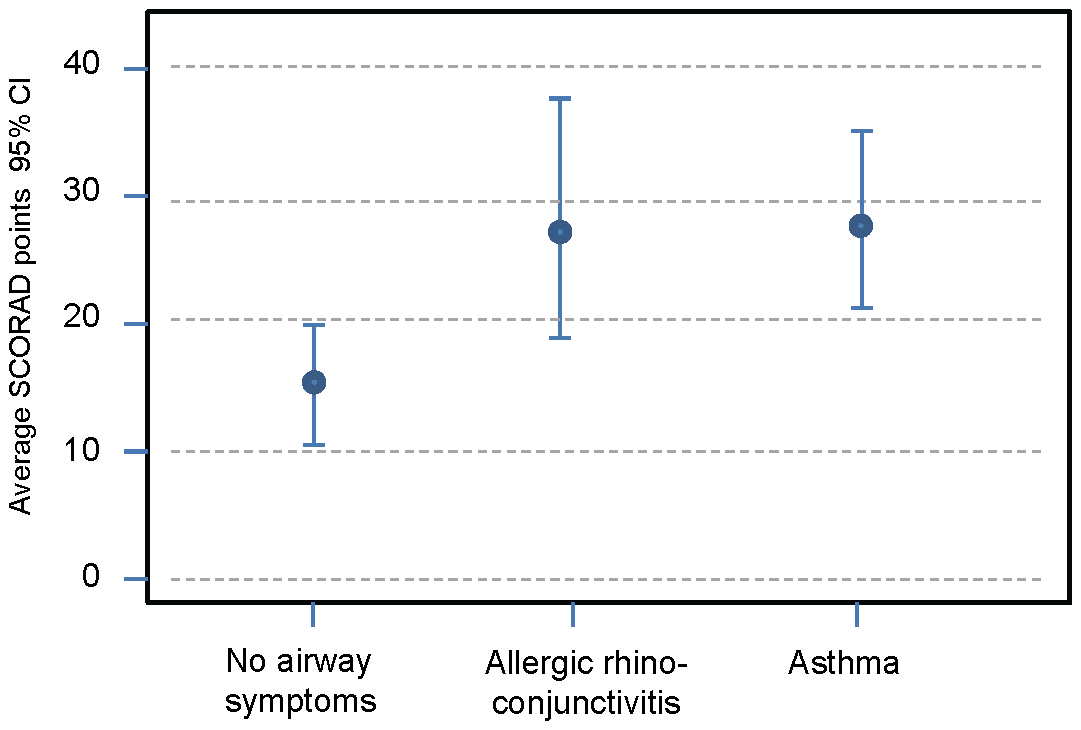
Average SCORAD points on inclusion for children with no airway symptoms, allergic rhinoconjunctivitis or asthma at 10 years.

**Figure 2:**
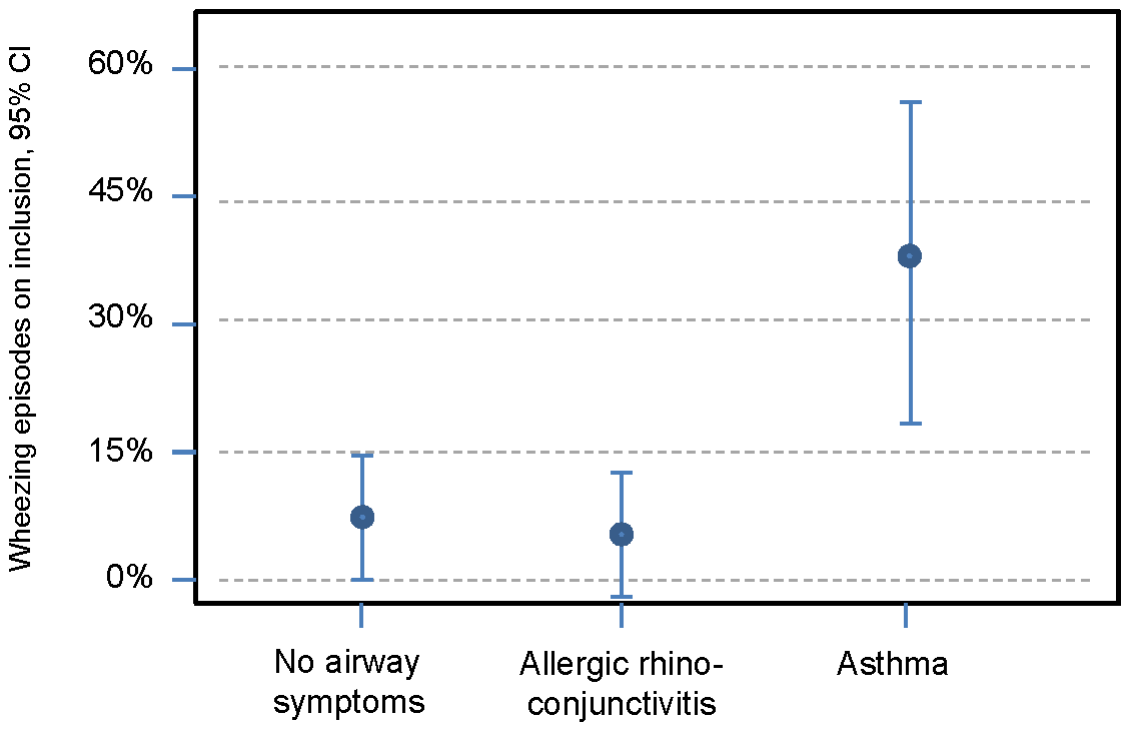
Percentage of children who had had at least one episode of wheezing before inclusion in the groups who had no airway symptoms, allergic rhinoconjunctivitis and asthma at 10 years.

**Figure 3:**
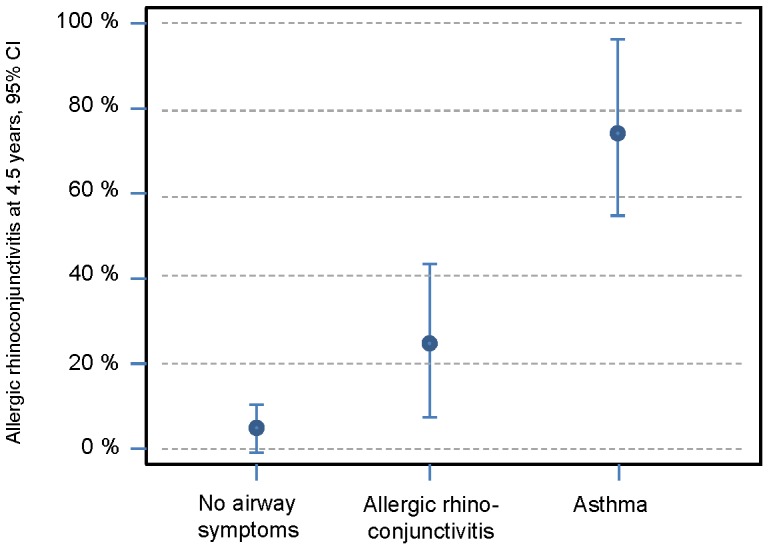
Percentage of children who had allergic rhinoconjunctivitis at 4.5 years among the groups with no airway symptoms, allergic rhinoconjunctivitis and asthma at 10 years.

**Figure 4:**
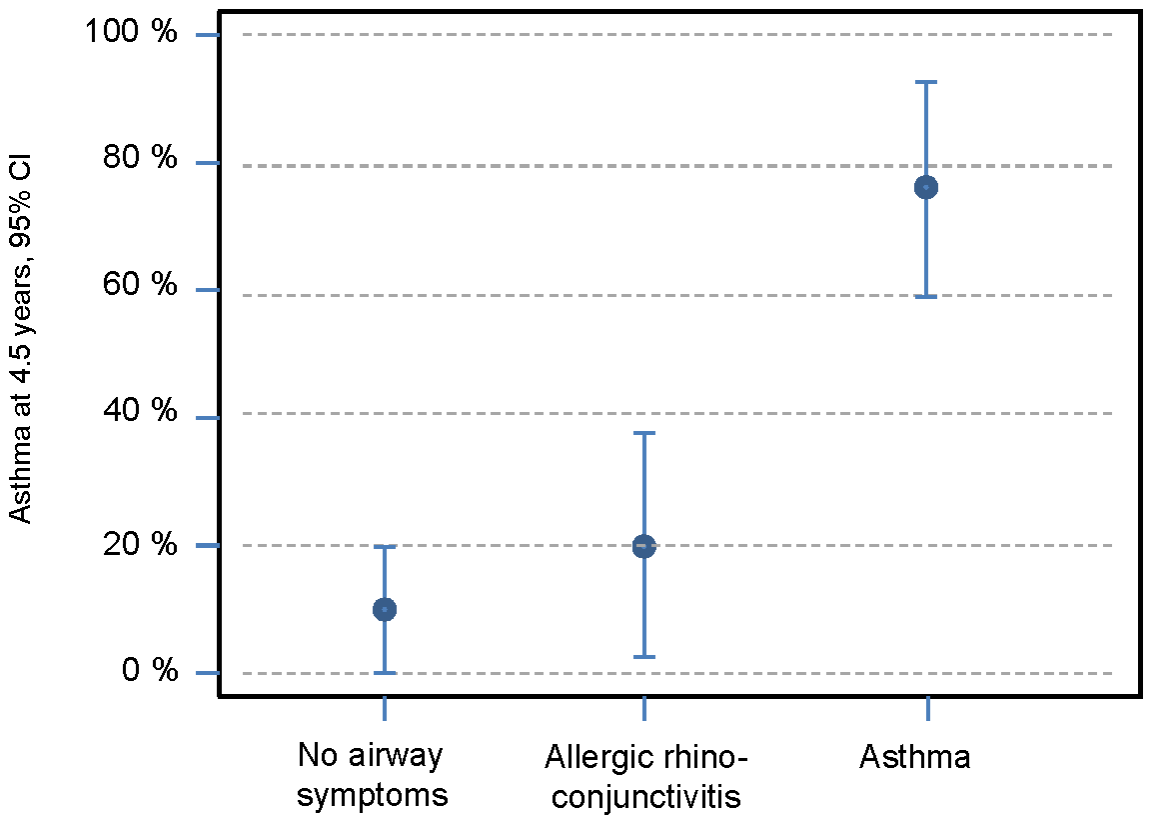
Percentage of children who were diagnosed with asthma at 4.5 years in the groups with no airway symptoms, allergic rhinoconjunctivitis and asthma at 10 years.

Binary logistic regression with ARC and asthma as covariates was used to calculate odds ratios (ORs) with 95% confidence intervals (CIs). Linear regression was used to study the relationship between different dependent scalar variables, ARC and asthma as independent variables. Significance tests of the regression coefficients, here referred to as B, together with estimates and accompanying CIs are presented. T test was used to analyze differences in SCORAD at the first visit between drop-outs and the study population. Differences combined with p values of <0.05 were considered statistically significant. Descriptive statistics were used to calculate means and confidence intervals for the groups with no airway symptoms, ARC and asthma seen in the Figures 1–4. All calculations were made with SPSS version 20 for Windows.

## Results

### Participation and summary of symptoms

At the follow-up at 4.5 years of age, 115 out of 123 children participated and several of them displayed more than one symptom. Of the 115, 60 still suffered from eczema, 26 had ARC, 37 had asthma and 35 had no symptoms of atopic disease at all. Ninety-four out of 123 completed the entire study until 10 years of age, of whom 60 continued to have eczema, 44 had ARC, 27 had asthma and 13 had no symptoms of allergy at all.

### Characteristics on inclusion and later risk of disease

At the first visit, the duration of breastfeeding in months, sensitization to cow's milk and egg, having furred pets at home, SCORAD points, eczema fulfilling Hanifin-Rajka criteria, episodes of wheezing and exposure to tobacco smoke were studied.

Higher SCORAD points on inclusion increased the risk of having ARC, B = 9.86 (95%CI 2.18–17.53; P = 0.01) and asthma, B = 10.17 (95%CI 2.82–17.52; P = 0.01) at 10 years of age.

For infants with eczema and wheezing at the first visit, the OR for being diagnosed with asthma at 10 years was 4.05 (95%CI 1.42–11.56; P = 0.01). Sensitization to egg correlated to the risk of an asthma diagnosis at 10 years of age, OR 2.59 (95%CI 1.00–6.67; P = 0.05). On inclusion, no statistically significant differences were found for fulfilled Hanifin-Rajka criteria, sensitization to milk, breastfeeding, having furred pets, or exposure to tobacco smoke.

### Characteristics at 4.5 years and later risk of disease

At 4.5 years of age, exposure to tobacco smoke, having furred pets at home, sensitization to milk, egg and aeroallergens, SCORAD points, eczema fulfilling Hanifin- Rajka criteria, ARC and asthma were studied.

For children with remaining sensitization to egg at 4.5 years of age, the OR for ARC at 10 years of age was 10.30 (95%CI 3.28–32.38; P = 0.00). For an asthma diagnosis at 10 years of age, the OR was 3.63 (95%CI 1.25–10.60; P = 0.02). Sensitization to aeroallergens at 4.5 years of age meant an OR of 4.70 (95%CI 1.50–14.75; P = 0.01) for ARC at 10 years of age and an OR of 6.21 (95%CI 1.83–21.06; P = 0.00) for asthma at 10 years of age. ARC at 4.5 years of age gave an OR of 11.28 (95%CI 3.61–35.28; P = 0.00) for asthma development but an OR of 3.05 (95%CI 0.88–10.60; P = 0.08) for only ARC at 10 years of age. For children diagnosed with asthma at 4.5 years of age, the OR for asthma at 10 years of age was 14.44 (95%CI 4.77–43.72; P = 0.00), whereas the OR for only ARC at 10 years of age was 1.08 (95% CI 0.34–3.47; P = 0.89).

No statistically significant differences were found for SCORAD points, having furred pets, remaining sensitization to milk, and fulfilled Hanifin-Rajka criteria at 4.5 years of age.

### Results at 10 years of age

Heredity, gender, exposure to tobacco smoke during the first 10 years, pets at home, sensitization to foods and aeroallergens, SCORAD, eczema fulfilling Hanifin-Rajka criteria, and values of exhaled NO before and after effort were studied at 10 years of age.

For children with eczema sensitized to aeroallergens at 10 years of age, the OR for ARC was 30.25 (CI 3.69-248.25; P = 0.00) and the OR for asthma was 5.84 (CI 1.65–20.71; P0.01). Higher levels of NO before effort resulted in B = 7.16 (CI 0.26–14.06; P = 0.04) for ARC and B = 13.25 (CI 6.61–19.89; P = 0.00) for asthma. For higher NO values after effort, the B for ARC was 7.76 (CI 0.85–14.66; P = 0.03) and for asthma, 13.26 (CI 6.35–20.16; P = 0.00).

The relation between eczema severity on inclusion and SCORAD points at 10 years of age was also studied. There was no significant difference in SCORAD points at 10 years of age between children with mild, moderate and severe eczema as infants.

Comparing the groups with no airway symptoms, ARC and asthma at ten years displayed no significant difference in positive family history of atopy (defined as at least one first-degree family member suffering from allergic symptoms), gender and fulfilled Hanifin-Rajka criteria.

To compare the representativity of the compliant cases with that of the original cohort, we also studied differences in SCORAD points on inclusion between the patients who had dropped out and those who remained in the study at 10 years of age. The drop-outs had a mean SCORAD point of 22.11, whereas those who remained had a mean SCORAD point of 21.47 on inclusion. The difference was not significant, P = 0.84.

## Discussion

In this prospective multicenter study we followed 123 children with eczema from infancy to 10 years of age to study factors that might influence and predict the risk of developing allergic airway symptoms.

Of the 123 patients, 94 completed the entire study. At the age of 10 years, 64% of the children still had problems with eczema, 47% had ARC, 29% had asthma, and only 14% had no symptoms of allergy. Therefore eczema in infancy must be considered a serious warning sign for lasting allergic problems rather than a transient condition, although eczema usually gets less severe with time [28]. Atopic disease during childhood often persists into adulthood [9]. The reported risk for children with atopic eczema of developing asthma varies between different studies. Our results are in line with several other studies [29]–[32]. Compared with the results from the Swedish birth cohort, BAMSE, which followed 810 children with infantile eczema to 12 years of age, the children in our study more often had persisting symptoms [33], [34]. This difference might be explained by the fact that the children in the present study who were diagnosed with eczema and referred to a pediatric clinic are likely to have had more severe symptoms than the children in BAMSE, whose were diagnosed with eczema based on questionnaire data.

We found that the risk of developing ARC and asthma before 10 years of age correlated with high SCORAD points during infancy. The SCORAD system has previously shown adequate validity and reliability to measure the severity of eczema [35], [36]. Our findings indicate that high SCORAD points in infancy could also be used to predict the risk of developing asthma and that severe eczema is more associated with allergic airway symptoms is than mild eczema. In contrast, high SCORAD points at the ages of 4.5 and 10 years were not associated with a higher risk of asthma or ARC when the patients were 10 years old. It has previously been shown that the severity of atopic dermatitis predicts the prognosis of eczema and the risk of developing other atopic manifestations [32], [37]. This study refutes the notion that severe eczema in infancy, classified according to SCORAD, is a predictor of a poorer prognosis of eczema later in childhood, as we did not find any correlation between high SCORAD points in infancy and high SCORAD points at 10 years of age. Nor did we find any correlation between high SCORAD points in infancy and remaining eczema at 10 years of age that fulfilled the Hanifin-Rajka criteria.

Wheezing in infancy is often transient, and infants tend to outgrow their breathing problems [16]. For our patients with eczema, however, wheezing meant a 4 times greater risk of having an asthma diagnosis at the age of 10 compared with infants affected by eczema only. This is not surprising as other atopic manifestations among wheezing infants are known risk factors for remaining asthma [38]. *C*hildren who fulfilled the criteria for asthma at 4.5 years had a 14 times higher risk of suffering from asthma at 10 years of age than had children with only eczema and no wheezing.

The children in our study who had symptoms of ARC at 4.5 years of age had a high risk of developing asthma. At 10 years of age, 67% of them were diagnosed with asthma, and the risk of developing asthma was 11 times higher than for 4.5 year olds without ARC. A strong association between ARC and asthma development was expected as Peroni et al found a strong correlation among 3–5 year olds [39]. Among adults, rhinitis is seen as an independent risk factor for developing asthma [40,41). Our study confirms the correlation between ARC and asthma for children with eczema. It is likely that eczema in infancy combined with early onset of ARC indicates a more severe allergic disease, which tends to lead to asthma development rather than ARC only.

Concerning food allergy, sensitization to hen's egg in infancy was correlated with a risk of developing asthma. Remaining sensitization to egg at 4.5 years of age was associated with both ARC and asthma development, confirming several other studies [37], [42]. In early childhood, sensitization to hen's egg has been associated with sensitization to airborne allergens [43]. At the age of 10 we found no correlation for egg sensitization, ARC and asthma. For our patients, sensitization to aeroallergens at 4.5 and 10 years of age was a strong predictor of allergic airway symptoms, as shown in earlier studies [44], [45].

Family history is a well-known risk factor for atopic diseases [46], [47]. Almost all patients in our study had a positive family history of atopy, with at least one first-degree family member suffering from allergic symptoms, which probably explains why heredity did not have any impact on the results. Parental smoking, before or immediately after birth, may not increase the risk of allergic sensitization in children, but it is a significant risk factor for recurrent wheezing in the first 1.5 years of life [48], [49]. In our study, only six children were exposed to tobacco smoke and almost no parents reported indoor smoking. This might explain why we were unable to find any correlation between tobacco smoke and airway problems in this study population.

To our knowledge, this is the only prospective study that has followed infants with eczema to the age of 10 with standardized methods such as SCORAD index and Hanifin-Rajka criteria to objectively describe the eczema, and with spirometric and NO tests for a reliable asthma diagnosis [50]. One concern was that following children for a long time could increase the number of drop-outs. It is plausible to assume that children who did not have any allergic symptoms would be less enthusiastic to continue the study and more likely to be found among our drop-outs, which could affect the result. This, however, has not been the case in other studies [33], [51], [52]. To minimize the risk of misinterpreting the results, we compared SCORAD points on inclusion for drop-outs with the rest of the study population and found no significant difference between the groups.

## Conclusions

This study indicates that high SCORAD points in infants with eczema are associated with a greater risk of asthma at 10 years of age. Children with eczema and wheezing episodes during infancy are more likely to get asthma later in life compared with infants with eczema only. Eczema in infancy combined with early onset of ARC appears to indicate a more severe allergic disease, which often leads to asthma development. The study shows that the progression from eczema in infancy to ARC at an early age and asthma later in childhood could support the relevance of the term “atopic march”, at least for children with a more severe allergic disease.
